# Sugarcane stem node detection with algorithm based on improved YOLO11 channel pruning with small target enhancement

**DOI:** 10.1371/journal.pone.0332870

**Published:** 2025-09-18

**Authors:** Chunming Wen, Leilei Liu, Shangping Li, Yang Cheng, Qingquan Liang, Kaihua Li, Youzong Huang, Xiaozhu Long, Hongliang Nong

**Affiliations:** 1 Guangxi Key Laboratory of Hybrid Computing and Integrated Circuit Design and Analysis, School of Artificial Intelligence, Guangxi University for Nationalities, Nanning, China; 2 Guangxi Zhuang Autonomous Region Intelligent Visual Collaborative Robot Engineering Research Center, Nanning, China; 3 Guangxi University for Nationalities, School of Artificial Intelligence, Nanning, China Guangxi Zhuang Autonomous Region Intelligent Visual Collaborative Robot Engineering Research Center, Nanning, China; 4 Guangxi University Engineering Research Center for Multimodal Information Intelligent Sensing Processing and Application, Nanning, China; 5 School of Physics and Electronic Information, Guangxi University for Nationalities, Nanning, China; 6 State Key Laboratory for Conservation and Utilization of Subtropical Agro-bioresources, Nanning, China; 7 Nanning Taiyin Technology Co., Ltd, Nanning, China; 8 Guangxi Agricultural Machinery Research Institute Co., Ltd, Nanning, China; 9 Nanning Taiyin Technology Co., Ltd, Nanning, China; Manipal Institute of Technology, INDIA

## Abstract

Sugarcane stem node detection is critical for monitoring sugarcane growth, enabling precision cutting, reducing spuriousness, and improving breeding for resistance to downfall. However, in complex field environments, sugarcane stem nodes often suffer from reduced detection accuracy due to background interference and shadowing effects. For this reason, this paper proposes an improved sugarcane stem node detection model based on YOLO11. This study incorporates the ASF-YOLO (Attentional Scale Sequence Fusion based You Only Look Once) mechanism to enhance the feature fusion layer of YOLO11. Additionally, a high-resolution detection layer, P2, is integrated into the fusion module to improve the model’s ability to detect small objects—particularly sugarcane stem nodes—and to better handle multi-scale feature representations. Secondly, to better align with the P2 small-object detection layer, this paper adopts a shared convolutional detection head named LSDECD (Lightweight Shared Detail-Enhanced Convolutional Detection Head), which can better deal with small target detection while reducing the number of model parameters through parameter sharing and detail-enhanced convolution. Using soft-NMS (non-maximum suppression) to replace the original NMS and combining with Shape-IoU, a bounding box regression method that focuses on the shape and scale of the bounding box itself, makes the bounding box regression more accurate, and solves the problem of the impact of detection caused by occlusion and illumination. Finally, to address the increased complexity introduced by the addition of the P2 detection layer and the replacement of the detection head, channel pruning is applied to the model, effectively reducing its overall complexity and parameter count. The experimental results show that the model before pruning has 96.1% and 53.2% mean average precision mAP50 and mAP50:95, respectively, which are 11.9% and 11.1% higher than the original YOLO11n, and the model after pruning also has 10.8% and 9.3% higher than the original YOLO11n, respectively, and the number of parameters is reduced to 279,778, and model size is reduced to 1.3MB. The computational cost decreased from 11.6 GFlops to 6.6 GFlops.

## Introduction

As the global demand for agricultural intelligence and automation increases, the combination of artificial intelligence and agriculture is gradually becoming one of the most important means to improve crop yield and quality. As an economically important crop, sugarcane is not only the main raw material for sugar production but also an important resource in the light industry, chemical industry, and energy sector. Currently, sugarcane is grown in more than 90 countries worldwide, covering an area of about 22 million hectares. Major sugarcane-producing countries include Brazil, India, China, Thailand, the United States, and Australia [1]. In China, Guangxi occupies 60% of the national planting area, and most of the sugar production [2], and the sugarcane industry has become an important economic industry in Guangxi. At present, in the sugarcane harvesting link, the whole stalk-type sugarcane combine harvester is limited by the hilly landscape environment in Guangxi; its operating conditions are high, and the operating effect is not fully realized. Even under the premise of the current rise of intelligent agriculture, the current field environment of sugarcane still requires a lot of manual work, especially in the identification and positioning of stalks, which still requires manual intervention, which not only increases labor intensity but also makes it challenging to improve operational efficiency. In the research and development of sugarcane intelligent harvesting technology, the identification of sugarcane stems and nodes under the complex light environment in the field is a problem that needs to be focused on.

With the rapid adoption of deep learning in agriculture, both one-stage detectors like YOLO [3] and two-stage methods like Faster R-CNN [4] have been applied to sugarcane stem node detection with promising results. Early efforts began in 2008, when Moshashai et al. [5] utilized diameter differences between stem nodes and internodes to initiate image-based detection. In 2010, Lu Shangping et al. [6] applied machine vision techniques using HSV color space thresholding and support vector machines for classification. Huang Yi-Qi et al. [7–8] later introduced grayscale and edge-based preprocessing methods, including local mean algorithms, to locate stem nodes. Recent years have seen the emergence of deep learning-based methods. In 2019, Li Shangping [9] proposed a YOLOv3-based real-time detection network, achieving 96.89% accuracy. Zhou et al. [10] (2020) enhanced RGB image analysis using median filtering and Sobel edge detection. Xie et al. [11] (2023) introduced a lightweight YOLOv5s variant by removing CBS and C3 modules, achieving high accuracy with only 2.6MB model size. In 2024, our group [12] proposed a field-adapted YOLOv7-based model by integrating SimAM attention and replacing some traditional convolutions with deformable layers, achieving 94.53% mAP and 92.41 F1-score.

In recent years, extensive efforts have been made to improve multi-scale feature learning and small object detection. Mask-Refined R-CNN (Regions with Convolutional Neural Networks) proposed by Zhang et al. [13] refines region-level features through a mask-guided mechanism and effectively preserves boundary details during multi-scale fusion, offering insights for enhancing small object detection accuracy. Lin et al. [14] introduced a feature disentanglement strategy in a one-stage detector, which models classification and localization tasks separately to mitigate task interference and improve both accuracy and robustness. The design principles of these works—multi-scale representation refinement and task-specific feature separation—align well with the motivation behind our lightweight ASF module [15], highlighting the potential of structurally optimized and robust detection frameworks in real-world agricultural applications.

In recent years, a growing number of advanced agricultural vision models have been proposed, covering various tasks such as fruit detection, weed recognition, and leaf disease classification. In fruit detection, Ma et al. [16] proposed an improved YOLOv7-tiny model for small apple detection under complex environments, demonstrating robustness to occlusion and illumination variations. In leaf disease identification, Sanida et al. [17] developed a two-stage transfer learning approach to accurately classify various tomato leaf diseases, achieving strong generalization performance across different datasets. To enhance detection performance in orchard settings, Zhang et al. [18] introduced a multi-scale feature adaptive fusion model that effectively improves the detection of small and partially occluded citrus fruits in real-time applications.

Although deep learning methods have achieved some success in recognizing sugarcane and stem nodes, most of the existing studies have focused on validation in indoor environments, and their detection capabilities in real complex field environments still need to be further explored. Fewer methods have been proposed for complex environments in the field, such as dust, cane leaf occlusion, etc., and there is a lack of a method that combines the two to achieve such high robustness and generalization as simultaneous detection of both indoor and outdoor.

While our method builds upon the YOLO family, it departs from incremental modifications in several key aspects tailored to the challenges of sugarcane stem node detection in complex field environments. First, we design a long-strip-aware shared convolutional detection head (LSDECD) to enhance the model’s ability to capture the spatial continuity and elongated structure of sugarcane stem nodes. Second, we introduce the Attentional Scale Sequence Fusion (ASF) module to improve multi-scale feature aggregation and robustness against occlusion and lighting variations. Third, we refine the Shape-IoU loss with a Soft-NMS-inspired formulation, allowing the model to be more sensitive to boundary shapes and morphological consistency—an important characteristic for structured agricultural objects like stem nodes. Furthermore, we apply LAMP (Layer-Adaptive Magnitude-based Pruning) to optimize the model structure, achieving significant compression without sacrificing accuracy. This pruning strategy is especially effective in agricultural scenarios with limited computation and power constraints. Combined with other lightweight design considerations, our model achieves deployment-friendly performance on edge devices, making it suitable for real-time applications in intelligent agricultural machinery.

## Methodology

### YOLO11 model

**Fig 1:**
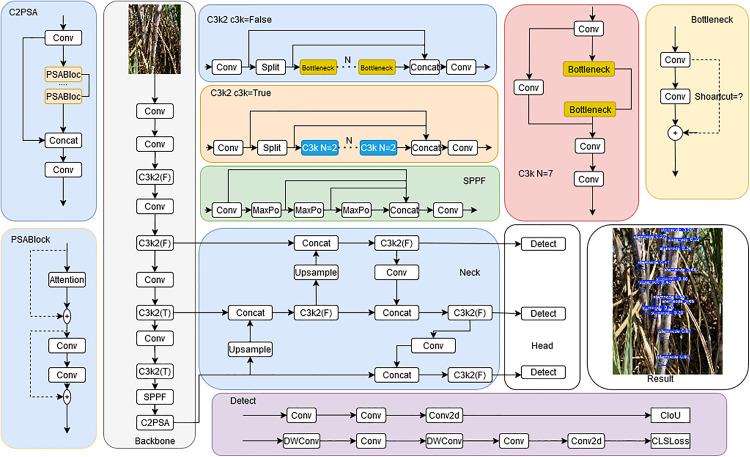
YOLO11 structure.

YOLO11 is a new generation of target detection algorithm proposed by the Ultralytics team in 2024 based on YOLOv8, and its core improvements include module reconstruction and structure optimization. First, the algorithm upgrades the C2f module of YOLOv8 to the C3k2 module: by dynamically controlling the c3k parameter, when c3k is activated, it replaces the original Bottleneck module with the CSP (Cross Stage Partial) Bottleneck with a smaller core to achieve a balance between lightweight and feature extraction efficiency. Meanwhile, the C2PSA (Cross-Stage Progressive Spatial Attention) mechanism is introduced to integrate the PSA (Pyramid Squeeze Attention) block in the C2f module, which enhances the ability to capture key features through attention weighting. Second, at the network structure level, YOLO11 adds a new C2PSA module after the SPPF (Spatial Pyramid Pooling Fast) module to optimize the gradient propagation and training stability by combining the multi-head attention and residual structure; the neck layer replaces the C2f uniformly with the C3k2 module to accelerate the process of multi-resolution feature aggregation and enhance the efficiency of cross-scale information fusion. In the detection head part, the algorithm adds two new deep convolutions (Depth-wise Conv), which utilize the independent computation of spatial dimensions to dramatically reduce the number of parameters and computational complexity while retaining the independence of cross-channel features. These improvements enable YOLO11 to balance detection accuracy and computational efficiency in complex scenarios, which is especially suitable for the needs of field environments with strong background interference and changing light in sugarcane stem node detection. The network structure of YOLO11 is shown in Fig 1.

### Improved attention scale sequence with new small target detection layer

Although the YOLO11 algorithm performs well in common task scenarios (e.g., pedestrian and vehicle detection), there are still many problems when it is directly applied to sugarcane stem node recognition in complex environments: for example, in real sugarcane environments, sugarcane grows densely and busily, the stem nodes are small in size and numerous in number, and to a certain extent, the presence of other sugarcane leaves and roots that are obscured or overlapped can lead to missed and false detection situations. Since sugarcane stem nodes usually belong to small targets in the pictures with small pixel ratios, the neck network of YOLO11 extracts feature information in 80 × 80, 40 × 40 and 20 × 20 pixel dimensions through the downsampling process and realizes the fusion of multi-scale information through the upsampling strategy. However, the method ignores the tiny-sized sugarcane stem node features, resulting in difficulties in recognizing these small targets at long distances.

**Fig 2:**
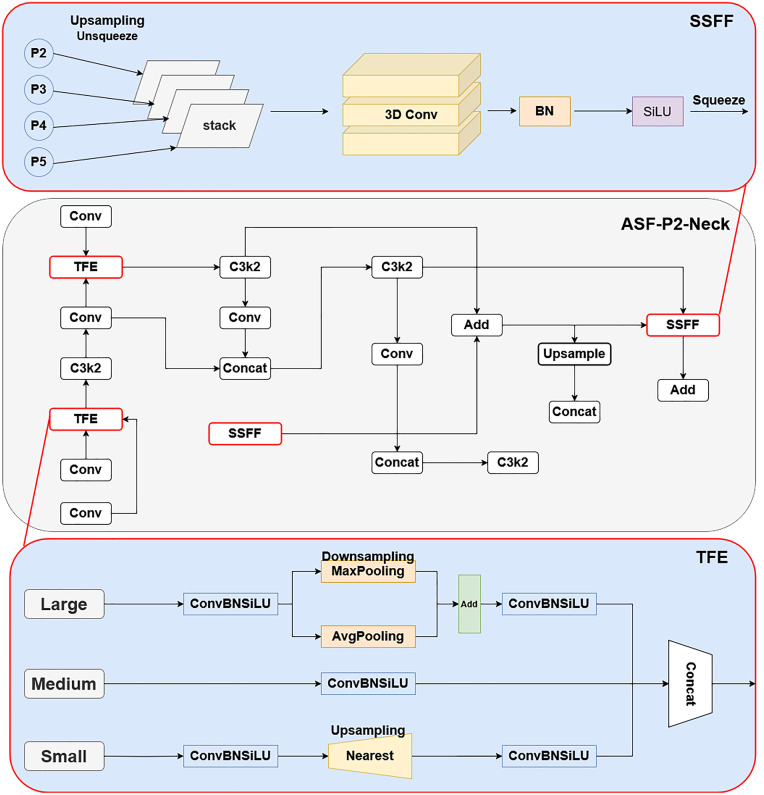
SSFF and TFE structural diagrams.

In order to address the above problems, this paper introduces an additional up-sampling layer at the neck layer to create a 160 × 160 pixel P2 detection layer. The detailed procedure of this improvement is shown in Fig 2. In order to maximize the detection of sugarcane stem nodes, the feature information is usually reduced after several layers of downsampling during feature extraction by the convolutional neural network. For example, when the step size is 16, the target area of 32 × 32 pixels is only 2 × 2 pixels in the feature map, and the effective area for detecting small objects cannot be recognized. Moreover, as the number of network layers deepens, small targets’ feature information and position information are gradually lost, which is not conducive to target localization. In convolutional neural networks, high-level feature maps have large sensory fields and rich semantic features but lose a large number of spatial features. On the contrary, the shallow feature map has a smaller receptive field [19], but it has high spatial resolution and accurate target location, which is suitable for small object detection, so a high-resolution feature layer (P2) is added to the original model to better utilize the shallow details and location information.

To further enhance the multi-scale feature fusion capability, this paper introduces the ASF structure into the Neck layer of YOLO11 to cope with the task of sugarcane stem node detection in the complex environment in the field. ASF is a target detection and segmentation framework that combines spatial attention with multi-scale features, and its core idea is to dynamically fusing feature maps from different layers of the backbone network.

In this paper, we add the P2 feature layer on top of the ASF architecture to comprehensively capture the multi-scale information of the target and form a new feature fusion architecture. The architecture mainly consists of the SSFF (Scale Sequence Feature Fusion Layer) module and the TFE (Triple Feature Encoder) module, which complement each other in global semantics and local detail modeling. The SSFF module aims to efficiently fuse the feature maps of P2, P3, P4, and P5, which capture different spatial scales covering various sizes of sugarcane in the dataset due to the shooting angle of the stem node targets; in SSFF, the feature maps of P2, P3, P4, and P5 are normalized to the same size and then stacked together as inputs to a three-dimensional (3D) convolution to combine multi-scale features.

The TFE module aims to enhance the detection of small targets in dense sugarcane stem nodes by splicing three different sizes of features, large, medium, and small, in the spatial dimension in order to capture detailed information about small targets. The detailed feature information of the TFE module is subsequently integrated into each feature branch through the PANet (Path Aggregation Network) structure. The TFE module aims to model fine-grained details across small, medium, and large receptive fields. It slices features at three spatial scales and fuses them via concatenation followed by convolutional layers. The final enhanced feature is:


$$F_{TFEX}=Conv(Concat(F_{l},F_{m},F_{s}))$$ (2–1)

Wherein, F_TFEX_ denotes the feature map output by the feature extraction module. F_l_, F_m_, and F_s_ correspond to the feature maps of large, medium, small size targets, respectively.

In order to solve the multi-scale problem of sugarcane stem nodes due to the shooting angle, existing literature uses the feature pyramid [20] structure for feature fusion, which usually merges the pyramid features using summation or concatenation. However, various feature pyramid networks must effectively utilize the correlation between all pyramid feature maps. SSFF better combines the high-dimensional information of the deep feature maps with the detailed information of the shallow feature maps. The image dimensions change during the downsampling process, but the scale-invariant features remain unchanged. The scale space is constructed along the image axes to represent not only a single scale but also a range of scales that an object may have. Although details may be lost in blurred images, structural features of the image can be preserved. Eqs (2–2) and (2–3) can obtain the scaled image input to SSFF:


$$F_{\sigma }(w,h)=G_{\sigma }(w,h)\times f(w,h)$$ (2–2)

$$G_{\sigma }(w,h)=\frac{1}{2\pi \sigma^{2}}e^{\frac{-(w^{2}+h^{2})}{2\sigma^{2}}}$$ (2–3)

Where f(w, h) denotes a 2D input image with width w and height h, $F_{\sigma }$ (w, h) denotes the smoothing process produced by f(w, h) by performing a series of convolution operations on the 2D Gaussian filter $G_{\sigma }$ (w, h). σ denotes the scale parameter that determines the standard deviation of the 2D Gaussian filter used in the convolution process. The SSFF module performs multi-scale feature aggregation by normalizing feature maps from different levels (P2, P3, P4, P5) to the same resolution and stacking them along the channel dimension. These are then processed using 3D convolution to capture inter-scale dependencies. The fused feature $F_{SSFF}$ can be expressed as:


$$F_{SSFF}=Conv3D(Concat(Resize(F_{P2}),Resize(F_{P3}),Resize(F_{P4}),Resize(F_{P5})))$$ (2–4)

where $F_{p_{i}}$ represents the feature map from level $P_{i}$, and Resize denotes upsampling/downsampling to a unified resolution.

The information from the TFE module is subsequently integrated into each feature branch through the PANet structure and combined with the multi-scale information from the SSFF module into the P3 branch. Three large, medium, and small feature maps with the same dimensions are convolved once and then spliced in the channel dimension. Building upon YOLO11, we reconstruct the Neck by integrating the concept of ASF, introduce a shallow P2 path, and optimize the feature fusion process. This approach aims to enhance small object detection performance with a lighter architecture and reduced complexity. The specific improvements are illustrated in Fig 2.

### Co-optimizing lightweight shared convolutional detection heads for small object detection

Aiming at the shortcomings of the traditional YOLO single-scale prediction structure in multi-scale feature utilization, YOLOv11 significantly enhances the multi-scale feature extraction capability through the introduction of improvements such as the C3k2 module and the C2PSA module, thus better adapting to the task of target detection in complex scenes. In this paper, a Lightweight Shared DEConv (the detail-enhanced convolutio) Detection Head [21] (LSDH) is designed to co-optimize with the added P2 feature layer. This detection head uses Group Normalization (GN) instead of the traditional Batch Normalization (BN) [22], which is especially suitable for small-batch training scenarios. Group normalization effectively improves the stability of model training and further optimizes the localization and classification performance by grouping feature channels to compute the normalization.

Since the introduction of the P2 layer makes the number of model parameters increase, in order to reduce the parameter size and computation of the detection head, LSDECD adopts a parameter-sharing strategy to integrate the original four feature extraction operations into a single process and adjusts the size of the feature map through the scale module. At the same time, Detail-Enhanced Convolution is introduced instead of the conventional convolution to improve the expressiveness and generalization of the feature extraction. DEConv includes five parallel convolutions: four differential convolutions and one regular convolution are included in the five parallel convolutions, which are used for feature extraction in parallel. Central Differential Convolution (CDC), Angular Differential Convolution (ADC), Horizontal Differential Convolution (HDC), and Vertical Differential Convolution (VDC) are used to integrate traditional local descriptors into the convolutional layer, thereby enhancing representation and generalization capabilities. In difference convolution, the differences of pixels in an image are first computed and then convolved with a convolution kernel to generate an output feature map; by designing a difference computation strategy for the pixels, a priori information can be displayed and encoded into a convolutional neural network (CNN). These convolutions are used for feature extraction and learning to enhance representation and generalization capabilities.

**Fig 3:**
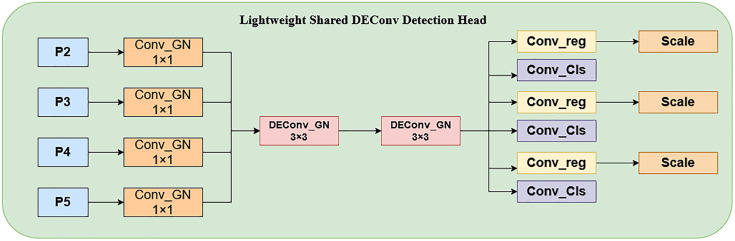
LSDED structure.

**Fig 4:**
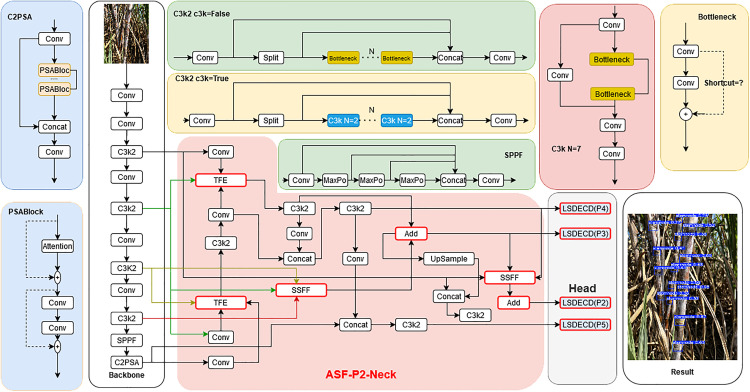
Improved structure of YOLO11.

The structure of the LSDECD detector head is shown in Fig 3, and the overall improved structure is shown in Fig 4, where the feature fusion layer is modified based on the original YOLO11, and the TFE, SSFF, and Add modules are introduced, replacing the original detector head with the LSDECD detector head, all of which are marked with a red box in the figure:

### Loss function improvement

**Fig 5:**
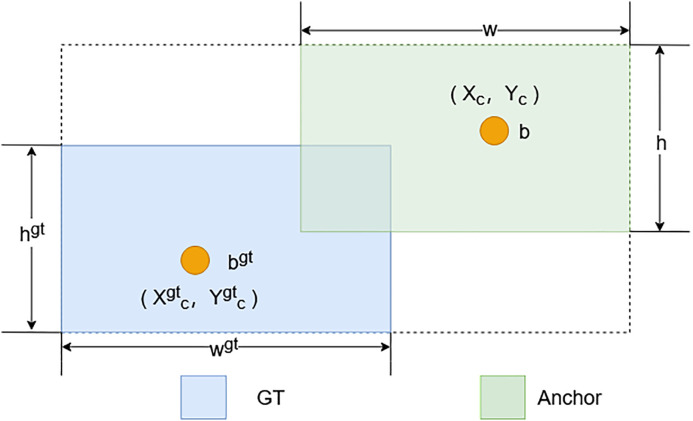
Shape-IoU structure diagram.


For the field environment, the detection effect is poor due to factors such as light and sugarcane leaf occlusion, and the sugarcane stem nodes also belong to the target of intensive detection. The Shape-IoU [23] loss function not only takes into account the geometrical relationship between the predicted frame and the actual frame but also integrates the shape and scale factors of the bounding box. Their IOU values may still differ for equal-sized bounding box regression samples despite having the same bias or shape bias. In addition, the bias or shape deviation in the direction of the short side of the bounding box has a more significant effect on the IOU values. Among the same-shaped bounding box regression samples, the IOU values of the smaller-size samples are more likely to be affected by the shape of the true box (GT). The formula for Shape-IoU can be derived from Fig 5, and its calculation is defined as follows:

$$IOU=\frac{\left|B\cap B^{gt}\right|}{\left|B\cup B^{gt}\right|}$$ (2–5)

$$\omega \omega =\frac{2\times {\left(\omega^{gt}\right)}^{scale}}{{\left(\omega^{gt}\right)}^{scale}+{\left(h^{gt}\right)}^{scale}}$$ (2–6)

$$hh=\frac{2\times {\left(h^{gt}\right)}^{scale}}{{\left(w^{gt}\right)}^{scale}+{\left(h^{gt}\right)}^{scale}}$$ (2–7)

$$ds\tan ce^{shape}=hh\times \frac{{\left(x_{c}-x_{c}^{gt}\right)}^{2}}{c^{2}}+\omega \omega \times \frac{{\left(y_{c}-y_{c}^{gt}\right)}^{2}}{c^{2}}$$ (2–8)

$$\Omega^{shape}=\sum {\mathrm{\backslash nolimits}}_{t=\omega ,h}{\left(1-e^{-\omega_{t}}\right)}^{4}$$ (2–9)

$$\omega_{\omega }=hh\times \frac{\left|\omega -\omega^{gt}\right|}{max\left(\omega ,\omega^{gt}\right)}$$ (2–10)

$$\omega_{h}=\omega \omega \times \frac{\left|h-h^{gt}\right|}{max\left(h,h^{gt}\right)}$$ (2–11)

Following Bodla et al. [24], we adopt the linear variant of Soft-NMS, which decays the confidence scores of overlapping boxes rather than eliminating them. Unlike traditional NMS that uses hard thresholding, Soft-NMS ensures that adjacent but distinct stem nodes are retained. First, the standard Non-Maximum Suppression (NMS) was replaced with Soft-NMS, which adjusts the suppression score using a continuous decay function rather than hard thresholding. This allows nearby overlapping boxes with slightly lower confidence to be retained, mitigating missed detections of dense and adjacent stem nodes.

The score update follows the linear decay version of Soft-NMS as in Eq (2–12):


$$s_{i}=s_{i}\cdot \left(1-IoU\left(M,b_{i}\right)\right)\;,\;if\;IoU\left(M,b_{i}\right)>Nt$$ (2–12)

Where $s_{i}$ is the score of box $b_{i}$, M is the highest scoring box, and Nt is the threshold. We set Nt = 0.5.

Second, we replace the IoU-based regression loss with a Shape-IoU loss, which introduces a shape-aware penalty to account for the width–height discrepancy, better capturing the anisotropic structure of sugarcane stem nodes. Shape-IoU is defined as:


$$ShapeIoU=\frac{A_{B\cap B^{gt}}}{A_{B\cup B^{gt}}}\cdot ShapePenalty$$ (2–13)

Where the ShapePenalty is:


$$ShapePenalty=exp\left(-\alpha \cdot \left|\frac{w}{h}-\frac{w^{gt}}{h^{gt}}\right|\right)$$ (2–14)

We set α = 1.5, balancing sensitivity to aspect ratio differences. This design was inspired by the shape-sensitive penalty mechanisms in attention modeling for forgery detection, as used in [25], where architectural attention is modulated by structural divergence. We adopt this spirit for spatial regularization in object shape regression. Finally, Shape-IoU is used to replace the original IoU in Soft-NMS, which can better distinguish the bounding box quality of slender targets. The following simultaneous formulas are then obtained:


$$s_{i}=s_{i}\cdot exp\left(-\frac{Shape-IoU{\left(b_{i},b_{M}\right)}^{2}}{\sigma }\right)$$ (2–15)

### Model lightweight

The addition of the high-resolution P2 detection layer and the replacement of the shared convolutional detection head increase the model parameters and complexity, making it difficult to be applied to resource-constrained application scenarios such as field environments.To facilitate deployment on resource-constrained devices, this paper employs LAMP (Layer-Adaptive Sparsity for Magnitude-Based Pruning) [26] to reduce the model size through structured pruning.

Traditional Magnitude-Based Pruning (MBP) usually uses a uniform pruning rate (i.e., all layers are pruned at the same rate) without considering the impact of different layers on the model performance, which may lead to poor pruning results. In this paper, we adopt a channel pruning approach using the LAMP algorithm to compute the channel importance score. Applying the concept of unstructured pruning to channel pruning enhances flexibility and adaptability, which enables accurate pruning of the model according to specific tasks and requirements. This flexibility enables optimization of the model structure according to different application scenarios and platform specifications.

First, LAMP is an unstructured pruning approach that computes a layer-adaptive sparsity score, which is then used to determine the appropriate sparsity levels across different layers and guide the magnitude-based pruning process. This approach balances sparsity and accuracy without the need to tune hyperparameters or perform complex computations. The fine-grained computation and pruning effects of unstructured pruning then motivate this paper to incorporate the LAMP score into the calculation of the importance score for structured pruning. Advanced channel pruning methods typically begin by sparsely training the bootstrap model, producing many scaling factors close to zero. Subsequently, connections corresponding to these near-zero scaling factors are removed, a process known as pruning, and the pruned model is then retrained to improve accuracy, a process known as fine-tuning. In this paper, the pruning scheme utilizes this score to simplify important score computation and channel pruning, thus avoiding extensive sparse training. This approach synchronizes the pruning process and retains the weighted connections with higher relative importance, thus maximizing the recovery of accuracy and minimizing the loss of accuracy during fine-tuning, with the score computed as shown in Equation (216):


$$Score(i;W):=\frac{{\left(W[i]\right)}^{2}}{\sum {\mathrm{\backslash nolimits}}_{j\geq i}{\left(W[j]\right)}^{2}}$$ (2–16)

Where |W[i]| denotes the weight tensor W mapped by index i and Score(i;W) denotes the LAMP score for the i-th index of |W[i]|. Consider a feedforward neural network with a fully connected layer whose weight tensor is a two-dimensional matrix, while for a two-dimensional convolutional layer, the corresponding tensor is a four-dimensional matrix. To define the score uniformly, each weight tensor is expanded into a one-dimensional vector, and for each expanded vector, the weights are assumed to be in ascending order of the index mapping, i.e., |W[i]| ≥ |W[j]| when i > j. The LAMP measures the relative importance of all the retained connections in the same layer and prunes those with lesser weights by a simple check, as expressed in Eq (217):


$${\left(W[i]\right)}^{2}>{\left(W[j]\right)}^{2}\Rightarrow Score(i;W)>Score(j;W)$$ (2–17)

In our implementation, pruning is performed in a layer-wise manner, without any additional sparse training. The pruning process is executed iteratively over 200 steps, where a regularization term is gradually increased using a linear decay schedule. During each iteration, the algorithm evaluates the importance of each convolutional channel by computing the LAMP score: the ℓ1-norm magnitudes of each channel’s weights are first sorted layer-wise, and the cumulative distribution is used to normalize the importance scores. Channels with the lowest LAMP scores are progressively pruned in each layer, ensuring a fine-grained local selection. The max_sparsity is set to 1.0 to allow full exploration of pruning potential in each layer, while the target speed_up ratio is fixed at 3.0 to constrain the total pruning intensity. The pruning regularization strength reg is set to 0.0005 and adjusted with a small increment at each step to gradually enforce sparsity.

**Fig 6:**
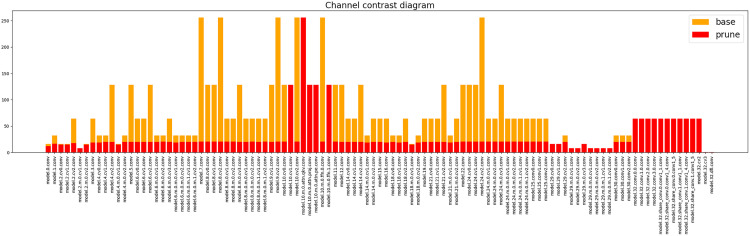
Before and after model channel pruning parameter counts.

The effect of pruning is shown in Fig 6, where the horizontal axis represents the network layer number, the vertical axis represents the number of channels, yellow is the number of parameters in each layer of the original model, and red is the number of parameters in each layer of the pruned model, and the number of channels in the feature fusion layer is drastically reduced, which suggests that there may be a high level of redundancy in the feature fusion layer, which contributes less to the task. The number of parameters can hardly be optimized in the detection head part, which shows that the most important structure affecting the size of the model is the detection head of the model, and the detection head is the key to detection and recognition. The channels of the backbone network and feature fusion layer are reduced by 84%, while the channels of the detection head are reduced by only 7%, so the detection head needs to keep more channels to maintain localization accuracy. After pruning, the number of model parameters is reduced by 90%, and FLOPs are reduced by 44%, but the mAP50 only decreases by 1.1%, indicating that pruning significantly improves inference speed while maintaining accuracy.

## Experimentation and analysis

### Dataset preparation

The dataset used in this study consists of 2,346 images. Among them, 1,618 images were collected from sugarcane fields in real-world outdoor conditions at the experimental site in Balian Village, Fusui County, Chongzuo City, Guangxi Zhuang Autonomous Region. The remaining 728 images were taken under controlled indoor conditions. These indoor samples were obtained from sugarcane collected at the Agricultural Science and Technology Innovation Base in Fusui County. After manual harvesting and leaf removal, the samples were photographed in a laboratory at Guangxi University for Nationalities. The outdoor images were captured by an Apple iPhone 13pro with a resolution of 3024 × 4032, while the indoor images were captured by a depth camera IntelD435i with a height of 1m and a resolution of 1920 × 1080 and were saved in jpg format. The images were randomly divided into 80% as the training set and 20% as the validation set. The dataset was then manually annotated using LabelImg by drawing bounding boxes around the sugarcane stem nodes in each image, with the annotations saved in YOLO text format.

### Experimental environment and evaluation indicators

In this study, model training was performed on a computer running the Ubuntu 20.04 operating system. The computer was configured with 16 virtual CPUs at 2.10 GHz. The GPU was an RTX 4090 (24 GB), and the experimental environment was based on Python 3.8 and CUDA 11.8. the relevant hyperparameters of the experimental model were set as follows: the model accepts an image with a resolution of 640 × 640 pixels as standard input, the initial learning rate is 0.01, and the optimizer uses SGD. Considering the variations in model size and training speed, the number of training batches is set to 16 per training batch, and the number of training iterations is 200.

On the test dataset, this paper uses several evaluation metrics to assess the performance of the training model. All samples are categorized into four types, i.e., True Positive Example (TP), False Positive Example (FP), True Negative Example (TN), and False Negative Example (FN). Precision (P) and recall (R) are defined based on the number of these four sample types. The metrics for evaluating the model in this paper mainly include average precision (mAP50, mAP50:95), model size, precision (P), recall (R), number of parameters, and FLOPs. These metrics can synthesize the model’s performance in the target detection task, including detection accuracy, efficiency, and complexity of the model. Specifically, the average precision is usually computed at different IoU thresholds, and the commonly used standard is mAP50 vs. map50:95. The inference speed of the model, on the other hand, is an important factor in assessing its usability in real-world applications, whereas the number of parameters and the computational FLOPS, in turn, directly affect the deployment of the model and the computational resource requirements. Among them, the formulas for precision, recall, and average precision are (3−1), (3−2), and (3−3), respectively:


$$P=\frac{TP}{TP+FP}\times 100\%$$ (3−1)

$$R=\frac{TP}{TP+FN}\times 100\%$$ (3−2)

$$mAP=\frac{1}{C}\int_{0}^{1}P(R)dR$$ (3−3)

### Comparison, ablation experiments and analysis of their results

To validate the independent and combined effectiveness of soft-NMS and Shape-IoU, ablation experiments were conducted sequentially on these improvements using our enhanced algorithm. Table 1 compares baseline (NMS + CIoU), Soft-NMS, Shape-IoU, and their combination.

**Table 1:** Comparative experimental results.

Method	mAP50/%	R/%
Baseline (NMS + CIoU)	93.2	89.7
+ Soft-NMS	95.2	90.6
+ Shape-IoU	94.7	91.1
+ Both	96.1	92.6

As shown in Table 1, the baseline model achieves 93.2% mAP50 and 89.7% recall. With the addition of Soft-NMS, the mAP:50 increases to 95.2% and the recall improves slightly to 90.6%, demonstrating that Soft-NMS effectively preserves overlapping detections, thus enhancing overall detection performance. Introducing Shape-IoU as the localization loss further improves both metrics, achieving 94.7% mAP:50 and 91.1% recall. Notably, when both Soft-NMS and Shape-IoU are applied simultaneously, the model achieves the best performance with a mAP:50 of 96.1% and recall of 92.6%. These results indicate that the combination of Soft-NMS and Shape-IoU provides complementary benefits, leading to more accurate and robust object detection, particularly in complex agricultural scenes involving dense and elongated targets.

**Fig 7:**
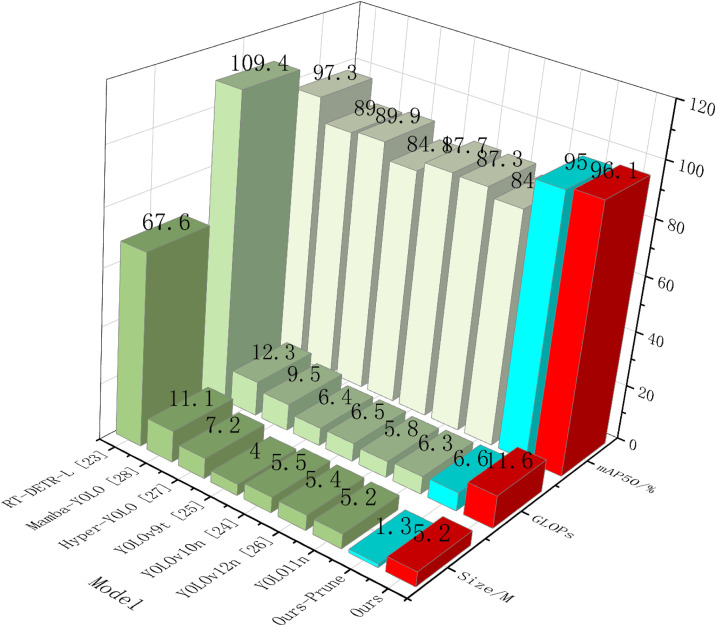
3-D comparison of key data across models.

In this paper, comparisons are made with other algorithms, including RT-DETR as well as the YOLO family of algorithms: RT-DETR-l, YOLOv8n, YOLOv9t, YOLOv10n, YOLOv12n, Hyper-YOLO, Mamba-YOLO, as well as YOLO11’s large models: YOLO11m, YOLO11s, YOLO11l, and all of them are trained under the improved loss function again validation comparison using the validation dataset. The comparison results are shown in Table 2 and Fig 7:

**Table 2:** Comparative experimental results.

model	mAP50/%	mAP50:95/%	parameters	GFLOPs	P/%	R/%	Size/M	Fps
RT-DETR-l [27]	97.3	51.8	33,529,539	109.4	94.9	94.3	67.6	41.5
YOLOv8n	83.9	42.3	2,684,563	6.8	88.2	77.7	5.3	103
YOLOv9t [28]	84.1	42	1,730,019	6.4	88.9	77.6	4.0	52.7
YOLOv10n [29]	87.7	40.4	2,265,363	6.5	81.5	81	5.5	88.9
YOLO11n	84.2	42.1	2,582,347	6.3	88.6	77.5	5.2	80.9
YOLO11s	85.7	44.3	9,413,187	21.3	90.8	78.5	18.3	86.1
YOLO11m	86.5	48.5	20,030,803	67.6	90.7	79.6	38.6	65.1
YOLO11l	85.3	45.3	25,280,083	86.6	89.9	78.3	48.8	42.9
YOLOv12n [30]	87.3	39.4	2,508,539	5.8	81.6	82.4	5.4	23.3
Hyper-YOLO [31]	89.9	41.6	3,620,979	9.5	85.3	84.4	7.2	32.3
Mamba-YOLO [32]	89.0	42.1	5,662,923	12.3	86.1	83.3	11.1	21.9
Ours	96.1	53.2	2,575,541	11.6	93.9	92.6	5.2	63.6
Ours-prune	95.0	51.4	279,778	6.6	92.3	90.7	1.3	78.9

The experimental results show that although RT-DETR-l reaches 94.9%, 94.3%, 97.3%, and 51.8% in the four important indexes of accuracy, recall, and mAP50. mAP50:95, respectively, of which the first three are in the leading position, its number of participants reaching ten million, and its vast GFLOPs are challenging to cope with the field scenarios. In contrast, the improved algorithm in this paper reaches 93.9%, 92.6%, 96.1%, and 53.2% in the four important indexes, respectively, and with only about one-tenth of the computational complexity and model size, it is also only slightly behind in terms of accuracy, and it even exceeds RT-DETR-l in mAP50:95, and compared with other algorithms of the YOLO series, it is in the leading position.

Moreover, our approach demonstrates superior performance over emerging models including YOLOv12n, Hyper-YOLO, and Mamba-YOLO in terms of both accuracy and model efficiency. Although Hyper-YOLO and Mamba-YOLO achieve competitive mAP50 scores (89.9% and 89.0% respectively), they still fall short of our method and have relatively larger parameter sizes (3.6M and 5.7M) and GFLOPs (9.5 and 12.3).

**Fig 8:**
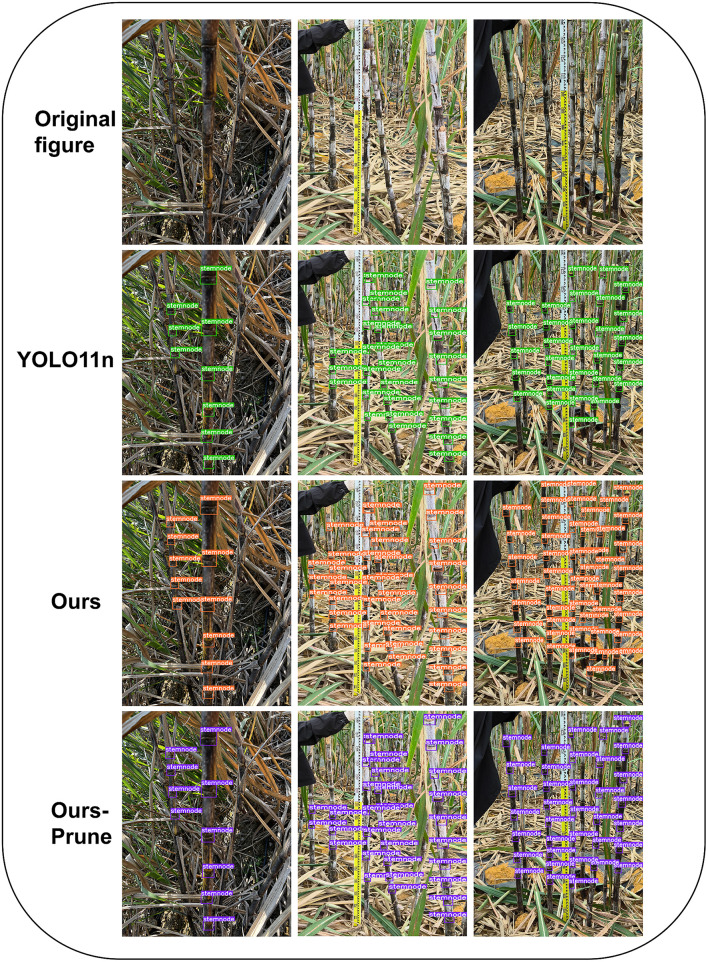
Comparison of recognition effects before and after improvement.

Meanwhile, in terms of lightweight, the pruned algorithm is one-tenth of the original YOLO11n in terms of the number of parameters, which is as low as 279,778, and the GFLOPs are only slightly larger than the YOLO11n model after adding a high-resolution detection layer, but the model size is only 1.3MB, which ensures the lightweight and at the same time, it still achieves the following results in terms of the four important indicators 92.3%, 90.7%, 95.0%, and 51.4%, which is also ahead of the other algorithms in the table. Before the improved loss function, the mAP50, as well as mAP50:95 using YOLO11’s CIoU loss function, both declined by 2.9% and 3.8%, respectively. The comparison effect between the improved algorithm and YOLO11n prediction is shown in Fig 8, which can be seen very intuitively through the predicted target box; the improved model predicts and recognizes a higher number of sugarcane stem nodes and higher accuracy than YOLO11n no matter in a small number of target scenes or in a complex scene, and still better than YOLO11n, even though the accuracy after pruning has decreased.

Inspired by the rigorous evaluation protocol adopted in Khan et al. [33], we structured our experiments to include extensive comparisons with state-of-the-art models, cross-validation, and statistical significance testing. To evaluate the robustness and generalization ability of the proposed model, we adopted a 5-fold cross-validation strategy. The dataset was randomly partitioned into five equal subsets. In each round, one subset was used as the validation set while the remaining four subsets served as the training set, the process was repeated five times. The experimental results are shown in Table 3. Subsequently, paired t-tests were performed on the five cross-validation sets. The test results demonstrated that mAP50 achieved a statistically significant improvement (t = 23.68, p < 0.0001), suggesting that the performance enhancement was not attributable to random chance. This method effectively reduces evaluation bias and ensures the reliability of experimental results.

**Table 3:** 5-fold cross-validation comparison results.

Number	YOLO11n mAP50/%	YOLO-SUG mAP50/%
1	84.7	95.7
2	84.1	96.4
3	84.0	95.3
4	85.2	95.7
5	84.6	94.1

**Fig 9:**
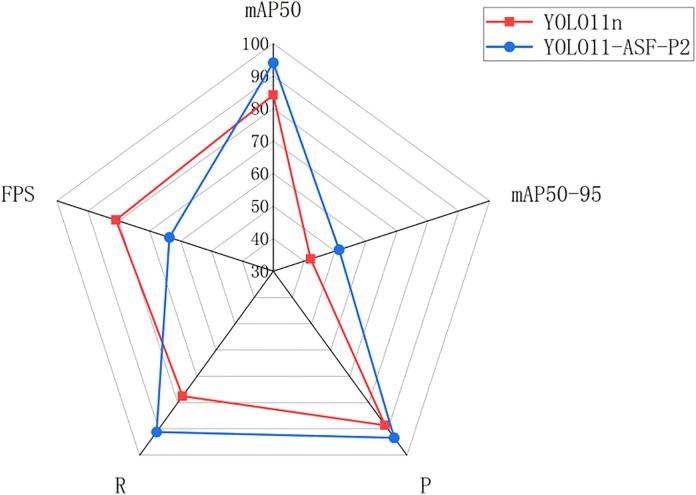
Comparison of key metrics before and after featuring fusion layer improvement.

To further validate the contribution of each improvement, ablation experiments were designed in this paper. The results are shown in Table 4 and Fig 9:

**Table 4:** Experimental comparison before and after modifying the feature fusion layer.

model	mAP50/%	mAP50:95/%	parameters	GFLOPs	P/%	R/%	Size/M	Fps
YOLO11n	84.2	42.1	2,582,347	6.3	88.6	77.5	5.2	80.9
YOLO11-ASF-P2	94.1	51.3	2,728,228	10.9	93.3	91.1	5.6	63.6

**Fig 10:**
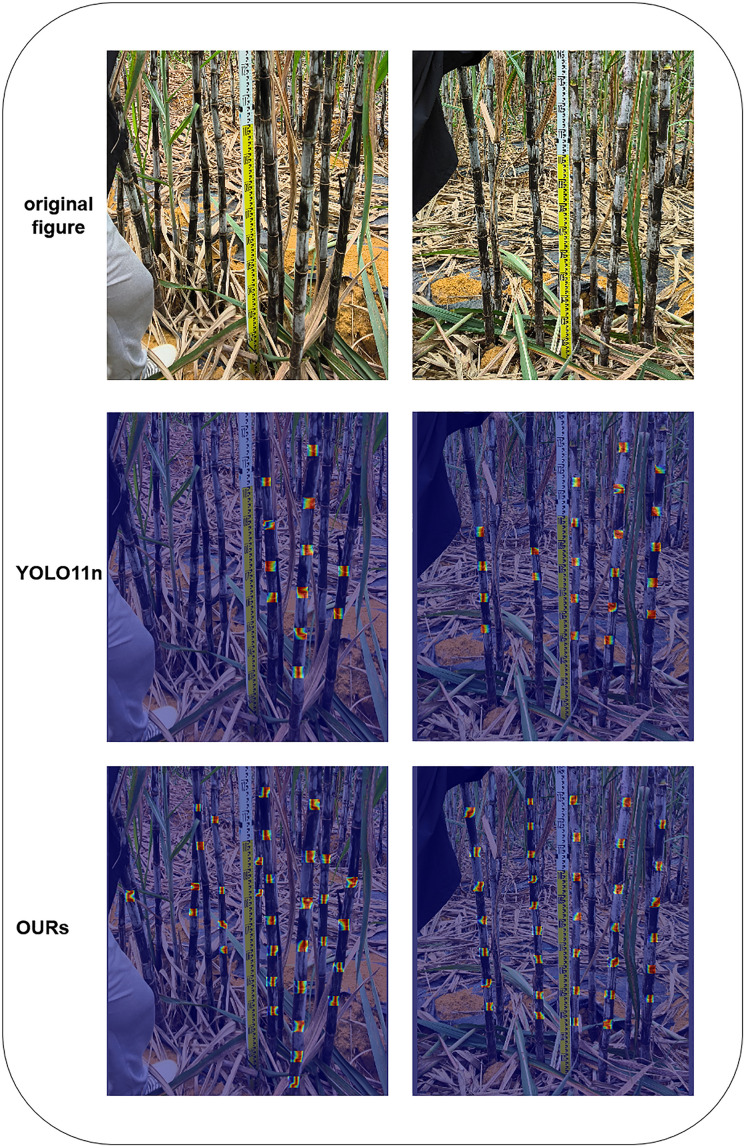
Comparison of thermograms before and after improving the feature fusion layer.

As can be seen from Table 4 and Fig 9, after the improved feature fusion layer, mAP50 is improved by 9.9%, and mAP50:95 is improved by 9.2%. The addition of the high-resolution small-target detection layer P2 to the feature fusion layer dramatically improves the model’s attention to detecting sugarcane stem nodes, but the amount of computation of the model is increased, which also makes the FPS decrease from 80.9 to 63.6. The effect of heat comparison graph is shown in Fig 10, comparing before and after the improved feature fusion layer; the redder color in the heat map indicates the more significant value, and the more heat box plots indicate that the model pays more attention to the sugarcane stem node target in the figure. It can be seen that the original version of the model in the target detection process of the stem node prediction is not accurate enough; there are leakage and false detection phenomena; after the improvement of the special fusion layer, the model in the localization of the stem node is more accurate and comprehensive, the average number of thermal box plots of a map are nearly 40% more than the original version of the thermal box plots.

To further validate the contribution of each component in the improved architecture, detailed ablation studies are presented in Table 5, which systematically quantifies the individual effects of the enhanced feature fusion layer, the P2 high-resolution detection module, and LSDECD detection head on model performance.

**Table 5:** Ablation study results.

Number	ASF	P2	LSDECD	mAP50/%	mAP50:95/%
NO.1				84.2	42.1
NO.2	√			89.7	42.4
NO.3		√		94.2	47.5
NO.4			√	89.1	45.3
NO.5	√	√	√	96.1	53.2

As shown in Table 5. The baseline model, without any enhancements, achieved an mAP50 of 84.2% and an mAP50:95 of 42.1%. Introducing the ASF module alone led to a significant increase in mAP50 (+5.5%) but brought only marginal gains in mAP50:95 (+0.3%), suggesting that ASF primarily enhances global semantic representation but is less sensitive to finer object boundaries. The addition of the high-resolution P2 detection head resulted in substantial improvements in both mAP50 (+10.0%) and mAP50:95 (+5.4%), confirming its effectiveness in detecting small and occluded targets. Similarly, integrating the LSDECD module provided consistent gains in detection performance, particularly in mAP50:95 (+3.2%), demonstrating its contribution to scale-aware feature extraction. When all three modules were combined, the model achieved the highest performance with an mAP50 of 96.1% and an mAP50:95 of 53.2%, indicating strong complementarity among the modules and validating the overall design. Based on the aforementioned improvements, the computational cost of the model reached 11.6 GFlops. Therefore, we pruned the model, and the pruning comparison results are shown in Table 6.

**Table 6:** Comparison of experiments before and after pruning.

model	mAP50/%	mAP50:95/%	parameters	GFLOPs	P/%	R/%	Size/M	Fps
Ours	96.1	53.2	2,575,541	11.6	93.9	94.0	5.2	63.6
Ours-Prune	95.0	51.4	279,778	6.6	92.3	90.7	1.3	78.9

**Fig 11:**
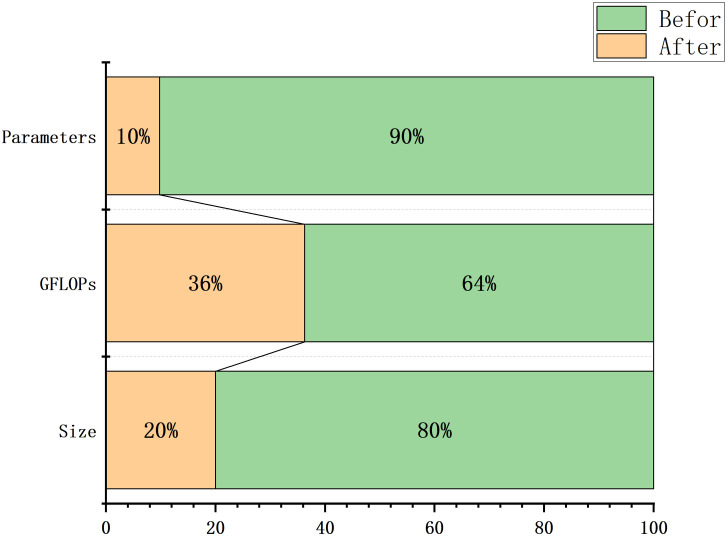
Comparison of key data before and after pruning.

As shown in Table 6 and Fig 11, after pruning, the model’s parameters decrease by nearly 90% (from 2.58M to 279K), while computation drops by 43% (5GFLOPs reduction). Despite slight mAP declines (1.1% in mAP50, 1.8% in mAP50:95), performance remains comparable to the YOLO11-SUG. The model size shrinks by 77% (5.2MB → 1.3MB), and detection speed improves by 15.3 FPS (63.6 → 78.9), enhancing real-time capability. These optimizations meet the requirements for field deployment in sugarcane plantations, balancing efficiency, accuracy, and hardware constraints.

### Scale-specific performance analysis

Our improved feature fusion layer method draws inspiration from the saliency-driven multi-scale perception framework proposed by Khan et al. [34], which emphasizes the role of attention-enhanced instinctive vision in improving small-object detection through saliency-aware feature refinement. Their work highlights that shallow, high-resolution feature layers are crucial for preserving spatial detail and local structure, especially for small and task-relevant targets. Building on this insight, we incorporate and adapt the Attentional Scale Sequence Fusion (ASF) module from ASF-YOLO into the neck of YOLO11, and further enhance it by introducing an additional P2 layer to better retain fine-grained spatial information. This attentional multi-scale fusion strategy enables the model to dynamically emphasize critical features across different scales, proving particularly effective in complex field environments where sugarcane stem nodes are frequently occluded or entangled with background vegetation.

To further validate the effectiveness of our proposed multi-scale fusion strategy, we based on the bounding box area size, we selected a subset of images from our datasets and classified the sugarcane stem node targets into small, medium, and large validation sets, containing 112, 162, and 177 samples respectively.

Experiments were conducted on these three validation sets in the same experimental environment using the baseline model YOLO11, the classic multi-scale detection method Efficientdet [35], and our improved YOLO11-SUG.

As shown in Table 7. Compared with YOLO11, our method achieves substantial improvements, particularly in small object detection, where the mAP50 increases from 82.4% to 94.2%. This demonstrates the effectiveness of introducing the ASF module and the additional P2 layer in preserving fine-grained spatial features. Furthermore, our model consistently outperforms EfficientDet, with slightly higher mAP50 on small objects (94.2% vs. 93.7%), and more evident gains on medium (96.8% vs. 94.3%) and large objects (97.7% vs. 96.4%). These results validate the robustness and adaptability of our architecture in complex field environments, especially for detecting dense and small-scale sugarcane stem nodes.

**Table 7:** Comparison of experiments before and after pruning.

model	Small mAP50/%	Medium mAP50/%	Large mAP50/%
YOLO11	82.4	87.6	90.2
Efficientdet	93.7	94.3	96.4
Ours	94.2	96.8	97.7

## Discussions

This paper proposes several enhancement strategies for YOLO11 to improve sugarcane stem node detection, particularly for small object detection in complex field environments. The addition of the high-resolution P2 detection layer improves recall and precision on small targets like stem nodes, especially in scenarios with occlusion and varying illumination. While the P2 layer increases computational complexity, channel pruning effectively mitigates this issue, reducing parameters to 279,778 and model size to 1.3MB.

The ASF feature fusion module enhances multi-scale representation, and the LSDECD detection head balances parameter sharing with detail enhancement, providing stronger representation for small targets without significantly increasing model size. In bounding box regression, Shape-IoU and Soft-NMS improve localization accuracy under occlusion and illumination challenges, though they introduce some computational overhead.

**Fig 12:**
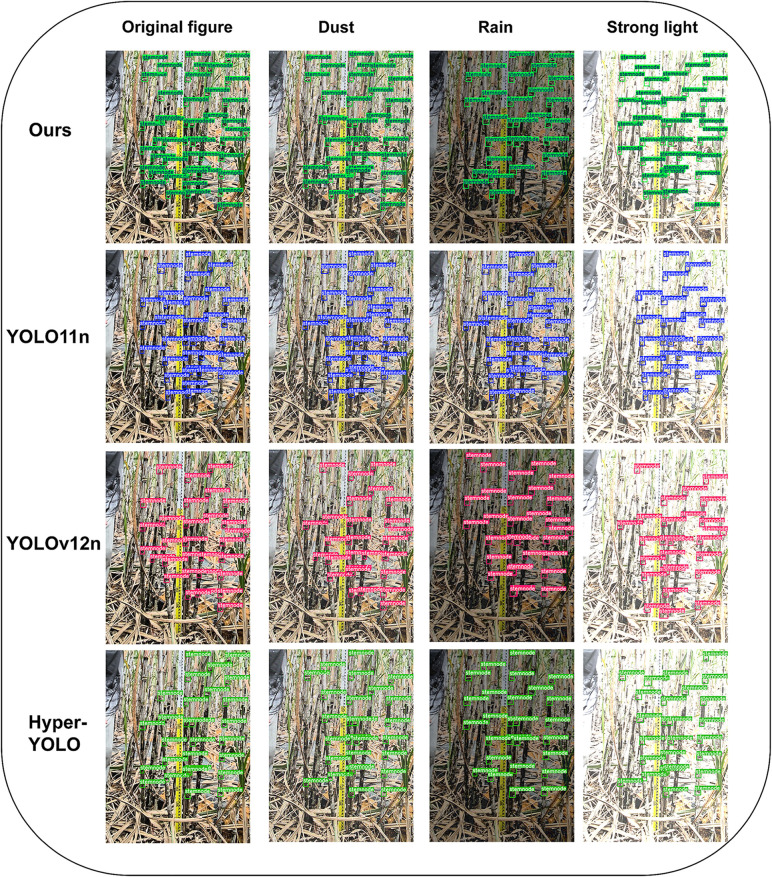
Comparison of prediction performance under extreme conditions.

Overall, the improvements in this paper show high utility in the sugarcane stem node detection task, specially making significant progress in small target detection and complex environment adaptation. However, the generalizability of these improvements still needs to be verified in a broader range of agricultural target detection tasks. To evaluate model robustness, data augmentation was performed on 659 images from the original dataset to simulate three extreme weather conditions: dust, rain, and strong light exposure. This expanded the augmented dataset to 1977 images, which were then divided into training and validation sets at an 8:2 ratio. Under the same experimental environment as the comparative experiments, the proposed model, YOLO11n, YOLOv12n, and Mamba-YOLO were sequentially trained and validated on this dataset. The experimental results are shown in Table 8, and the prediction comparison is illustrated in Fig 12.

**Table 8:** Comparative robustness evaluation under extreme conditions.

model	mAP50/%	mAP50:95/%	ΔmAP50/%	ΔmAP50:95/%
Ours	94.7	49.4	−1.4	−3.8
YOLO11n	81.3	37.1	−2.9	−5.0
YOLOv12n	83.8	37.4	−3.5	−2.0
Mamba-YOLO	85.6	37.9	−3.4	−4.2

While real-world multi-condition data collection is ideal but logistically constrained, our approach was inspired by Khan et al. [36], who emphasized enhancing feature robustness under visually challenging environments through attention-guided feature selection. Following this principle, we designed a comparative experiment to test the generalizability of our model against YOLO11n, YOLOv12n, and Mamba-YOLO under simulated complex conditions.

The experimental results demonstrate that, after being subjected to simulated extreme weather conditions, all four models exhibited a decline in both mAP50 and mAP50:95 metrics. However, our model experienced the smallest reduction in mAP50, and its decrease in mAP50:95 only slightly surpassed that of YOLOv12. Furthermore, our model maintained higher precision values compared to the other three models, which strongly suggests its robust performance.

Nevertheless, while the proposed improvements significantly enhance detection accuracy and robustness in field conditions, model deployment efficiency on edge devices remains a critical concern in real-world agricultural scenarios. Due to hardware constraints, real-world inference performance testing on platforms such as Jetson Nano or Raspberry Pi 4 was not conducted. However, to assess the model’s deployment feasibility, we referred to several representative benchmarking studies of YOLO-based models on edge computing devices [37–41].

Alqahtani et al. [37] analyzed the performance trade-offs between accuracy and latency on devices such as Jetson Nano and Raspberry Pi, showing that while SSD-MobileNetV1 provides low inference time, YOLO variants deliver better accuracy at acceptable latency. Feng et al. [38] demonstrated that YOLOv3-tiny and YOLOv4-tiny can achieve over 20 FPS on Raspberry Pi 4 with Intel NCS2 accelerators.

The large-scale benchmarking work YOLOBench [39] confirmed that lightweight YOLO variants with fewer than 1M parameters and sizes under 5MB typically achieve 20–30 FPS on Jetson Nano. More recent studies like FRYOLO [40] optimized YOLOv8 using pruning and Ghost modules to achieve 33 FPS on Jetson Orin Nano with minimal accuracy loss. Edge-YOLO [41] adopted a pruning edge-cloud collaboration strategy, achieving 26.6 FPS on embedded systems while maintaining high robustness.

Our pruned model, with only 279,778 parameters and 1.3MB in size, is significantly smaller than mainstream lightweight detectors such as YOLOv5n or YOLOv8n. Given the similar or higher resource demand of the models evaluated in, it is reasonable to infer that our model could also achieve real-time performance on edge hardware, especially when combined with inference optimizations such as quantization, Tensor-RT, or ONNX acceleration.

This indicates that the proposed model, while tailored for robust sugarcane stem node detection in challenging environments, also holds promise for practical deployment in resource-constrained agricultural edge systems, where low latency and compact model design are critical.

## Conclusions

In this paper, a series of improvements are proposed for the sugarcane stem node detection task, including the introduction of an ASF feature fusion layer, the addition of a high-resolution small-target detection layer P2, the adoption of a shared convolutional detection head LSDECD, the combination of Soft-NMS and Shape-IoU, and the optimization of the model complexity by channel pruning. Experimental results show that these improvements significantly enhance the detection performance and practical application value of the model.

Before pruning, the mAP50 and mAP50:95 of the improved model reaches 96.1% and 53.2%, respectively, which are improved by 11.8% and 9.1% compared with the original YOLO11n, which thoroughly verifies the superiority of the improved method in small target detection and multi-scale feature processing. The pruned model effectively reduces the number of parameters and computational complexity while mAP50 and mAP50:95 still improve by 10.8% and 9.3%, respectively, compared with the original YOLO11n, which indicates that the pruning strategy is able to significantly improve the inference efficiency while maintaining the model performance, and it provides good support for practical deployment.

In summary, the method proposed in this paper not only achieves significant improvement in detection accuracy but also effectively balances the relationship between model complexity and performance through channel pruning. These improvements explore feasible technical paths for agricultural intelligent detection tasks such as sugarcane stem node detection and have specific practical application value. Future research can further optimize the model structure, improve its robustness and adaptability in complex field environments, and provide specific technical references for the development of agricultural automation.
